# The role of Epstein–Barr virus in nasopharyngeal carcinoma

**DOI:** 10.3389/fmicb.2023.1116143

**Published:** 2023-02-09

**Authors:** Zhi Yi Su, Pui Yan Siak, Chee-Onn Leong, Shiau-Chuen Cheah

**Affiliations:** ^1^Faculty of Medicine and Health Sciences, UCSI University, Bandar Springhill, Negeri Sembilan, Malaysia; ^2^AGTC Genomics Sdn Bhd, Pusat Perdagangan Bandar, Persiaran Jalil 1, Bukit Jalil, Wilayah Persekutuan Kuala Lumpur, Malaysia

**Keywords:** nasopharyngeal carcinoma, Epstein–Barr virus, biomarker, therapeutic, diagnostic, screening

## Abstract

Nasopharyngeal carcinoma (NPC) is a metastasis-prone malignancy closely associated with the Epstein–Barr virus (EBV). Despite ubiquitous infection of EBV worldwide, NPC incidences displayed predominance in certain ethnic groups and endemic regions. The majority of NPC patients are diagnosed with advanced-stage disease, as a result of anatomical isolation and non-specific clinical manifestation. Over the decades, researchers have gained insights into the molecular mechanisms underlying NPC pathogenesis as a result of the interplay of EBV infection with several environmental and genetic factors. EBV-associated biomarkers were also used for mass population screening for the early detection of NPC. EBV and its encoded products also serve as potential targets for the development of therapeutic strategies and tumour-specific drug delivery. This review will discuss the pathogenic role of EBV in NPC and efforts in exploiting the potential of EBV-associated molecules as biomarkers and therapeutic targets. The current knowledge on the role of EBV and its associated products in NPC tumorigenesis, development and progression will offer a new outlook and potential intervention strategy against this EBV-associated malignancy.

## 1. Introduction

Epstein–Barr virus (EBV), formally known as human herpesvirus 4, is classified under the *Lymphocrytovirus* genus of the *Gammaherpesvirinae* subfamily within the *Herpesviridae* family. It was first discovered in Burkitt lymphoma cells during the 1960s (Young et al., 2016; Wong et al., 2022). Classified as a group I carcinogen, the virus is linked to 1.5% of all human malignancies and 1.8% of all cancer-related deaths, including Burkitt lymphoma, Hodgkin’s lymphoma, B-cell, T-cell, and NK-cell lymphoma, gastric carcinoma and nasopharyngeal carcinoma. Nasopharyngeal carcinoma (NPC) and gastric carcinoma accounted for 82% of EBV-attributed malignancies and 89% of deaths attributed to EBV-associated neoplasms. EBV is known to cause lifelong persistent infection asymptomatically in over 90% of the global population. Despite that, only a small proportion of the global community developed NPC, the incidences particularly displayed remarkable predilection towards certain populations and geographical areas. In endemic regions of NPC, EBV contributes to 95% of NPC incidences and 100% of NPC-related mortalities. In contrast, 20% of NPC incidences and 80% of NPC mortalities are attributed to EBV in low incidences areas (Khan and Hashim, 2014; Khan et al., 2020). Furthermore, NPC displays predominance in males, whereby the NPC incidences are two- to three-fold higher in men than women. The unique distribution of NPC cases implied the role of multiple factors, including host genetics and environmental factors that interplay with viral factors in NPC tumorigenesis (Chua et al., 2016; Chen et al., 2019).

NPC is histologically classified into keratinizing squamous cell carcinoma (type 1), nonkeratinizing squamous cell carcinoma (type 2), and undifferentiated carcinoma (type 3). The prevalence of EBV is 100% in type 2 and type 3 NPC, which is predominant in endemic areas (Chen et al., 2019). Currently, the Union for International Cancer Control or American Joint Committee on Cancer (UICC/AJCC) staging system is used for treatment guidance and prognosis prediction in NPC patients. The adoption of intensity-modulated radiotherapy alone or with chemotherapy for the treatment of NPC has achieved a favourable clinical outcome, with a decline in the age-standardised mortality rate over the decade. The 5-year overall survival (OS) rate is as high as 94% for patients diagnosed at an early stage, whereas a drastic decline in the 5-year OS rate (73.7%) is observed in patients with late-stage (stage III and IV) NPC (Jen et al., 2020). Nevertheless, owing to nonspecific symptoms of NPC, including headache, epistaxis, and facial pain, early detection of NPC remains a major challenge. Furthermore, 5 to 30% of NPC patients will develop post-treatment locoregional or distant recurrence (Li et al., 2014a; Lee et al., 2015; Tang et al., 2016; Jen et al., 2020; Yu et al., 2021b). Thus, diagnostic, predictive and prognostic biomarkers with high accuracy, precision, sensitivity and specificity are needed for the early diagnosis of NPC and prediction of treatment response of NPC patients. Furthermore, effective therapeutic strategies targeting the specific molecular targets in NPC could improve the clinical outcome of NPC patients. The current review discusses the oncogenic role of EBV in NPC and its translation into clinical application.

## 2. The pathogenic role of EBV in NPC

**Figure 1:**
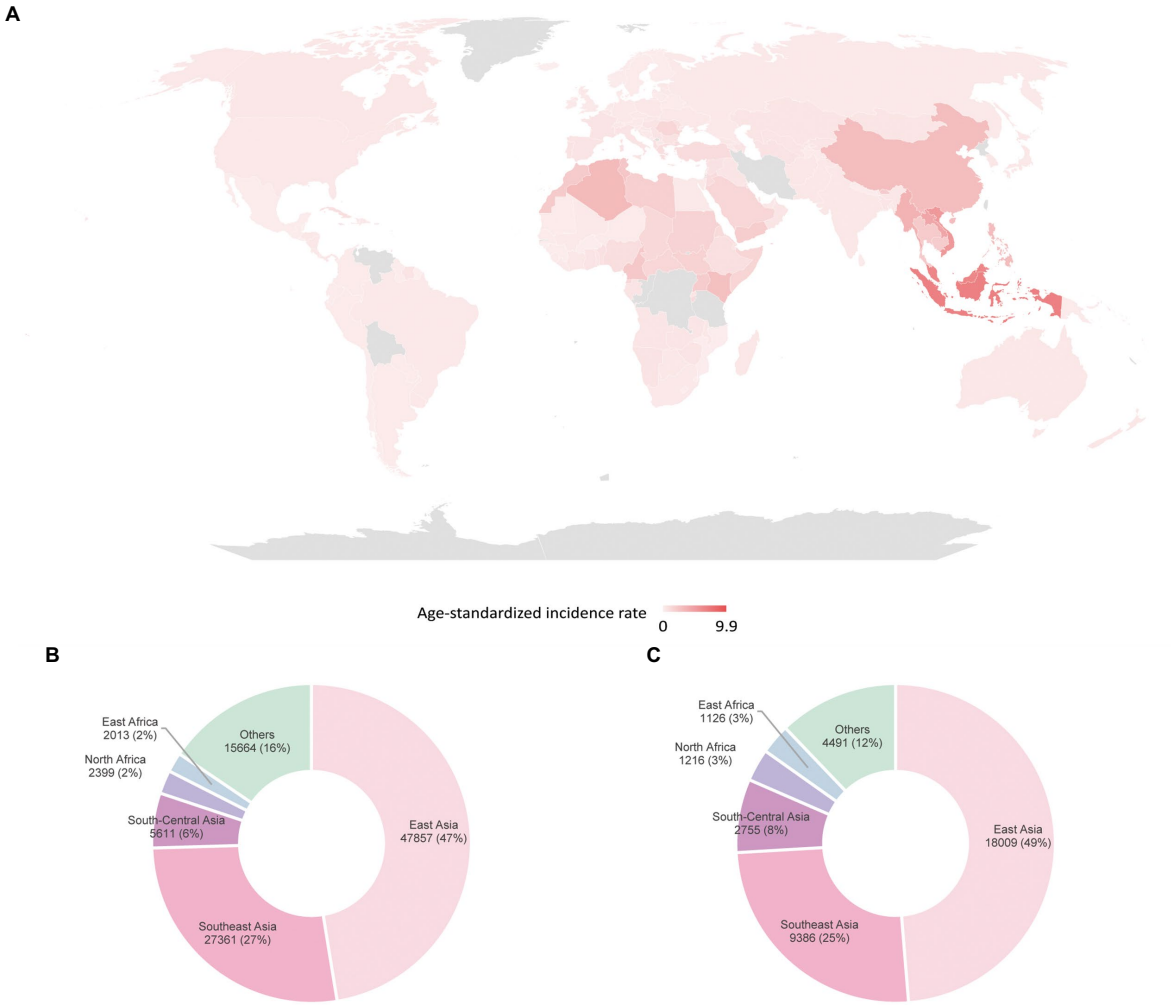
Global distribution of NPC estimated by GLOBOCAN in 2020 **(A)** age-standardised incidence rates **(B)** incidences in males **(C)** incidences in females (Sung et al., 2021).

**Figure 2:**
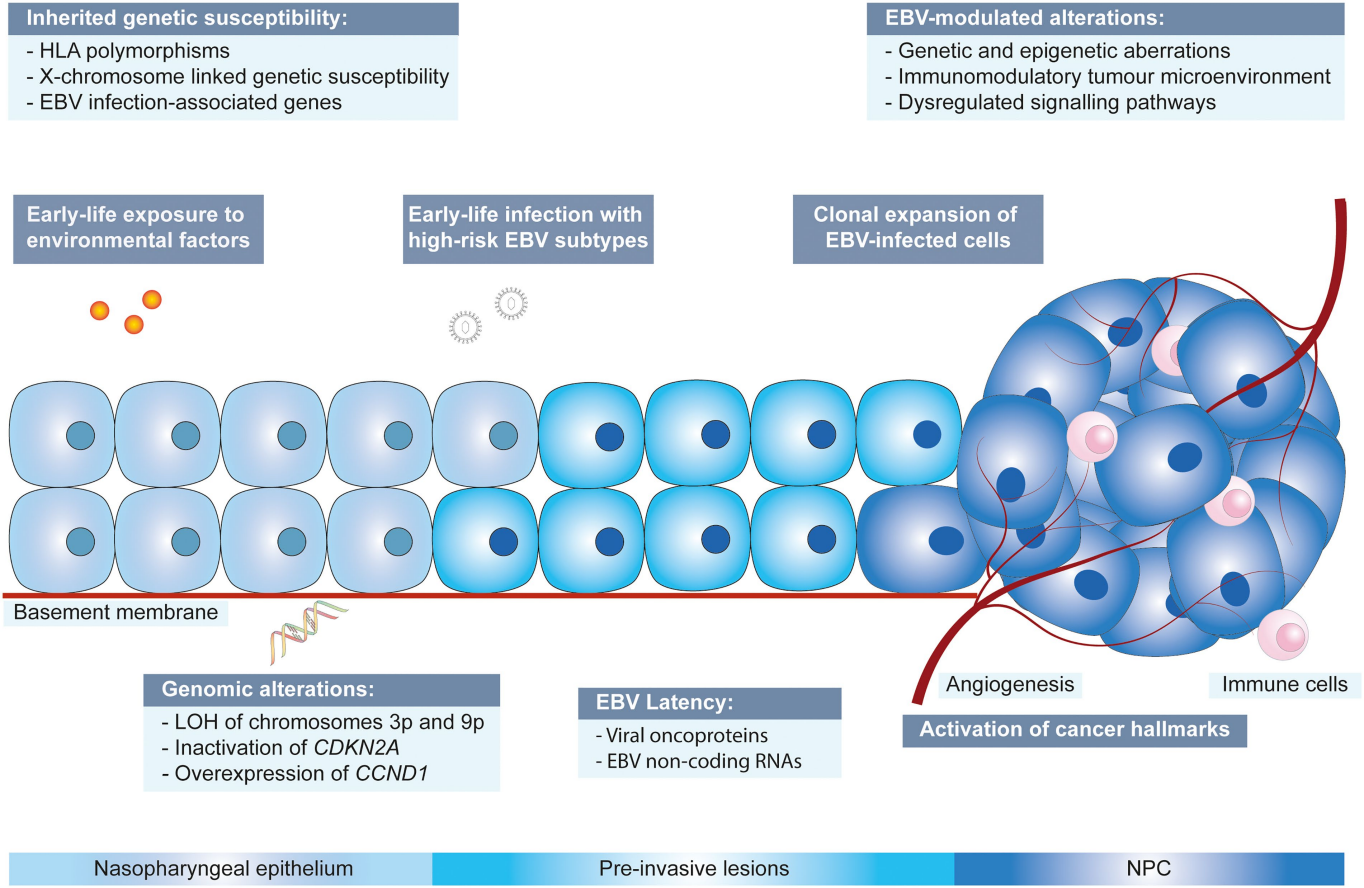
Multistep developmental model of NPC driven by collaboration between EBV, environmental factors and genetic susceptibility. CCND1: cyclin D1; CDKN2A: cyclin dependent kinase inhibitor 2A; EBV: Epstein–Barr virus; HLA: human leukocyte antigen; LOH: loss of heterozygosity.

The non-keratinizing carcinoma, accounting for the majority of NPC cases in the endemic regions, is strongly associated with EBV infection. The disease is characterised by an enigmatic geographical and ethnic distribution, wherein East and Southeast Asia accounted for over 70% of NPC global incidences, followed by South-Central Asia (6.3%), North Africa (2.6%) and South Africa (2.4%; Ferlay et al., 2020; Sung et al., 2021; Figure 1). The disease displays predilection towards some ethnic groups, including the Cantonese population residing the Southern China, the Bidayuh in Borneo, and the Inuits living in the Arctic (Tsao et al., 2014; Chua et al., 2016). The distinctive epidemiological pattern implied that NPC pathogenesis is a multistep process involving the interplay between EBV, host genetic predisposition, and environmental factors including tobacco smoking, dietary components, and occupational exposure. Over the decades, the implementation of advanced techniques contributed to deeper insights into the genomic and epigenomic landscape, the tumour microenvironment, and the pathogenic role of EBV in NPC. Figure 2 illustrates the pathogenic model of NPC, while Table 1 briefly summarises the pathogenic role of EBV-encoded products in NPC.

**Table 1:** Pathogenic role of EBV in NPC.

Pathogenic role of EBV	Viral products	Molecular mechanisms
Genetic instability in NPC	EBNA1	Disrupts PML NBs (Sivachandran et al., 2008); Upregulates DNMTs and PcG family proteins (Chen et al., 2021b)
	BALF3	Induce chromosome aberrations and DNA strand breaks (Chiu et al., 2014)
	LMP1	Represses DDB1-mediated nucleotide excision repair (Chen et al., 2008); impairs Chk1 activation (Deng et al., 2012); upregulates DNMTs (Tsai et al., 2006)
	miR-BART5-5p, 7-3p, 9-3p, 14-3p	modulate *ataxia telangiectasia mutated* (*ATM*) gene activity (Lung et al., 2018)
Inflammatory response	EBERs	Enhances TNF- α levels (Li et al., 2015)
	LMP1	Upregulates MIP-1α and -1β (Lai et al., 2010)
Evasion from immune surveillance	EBNA1	Promotes CCL20- and transforming growth factor (TGF)-β-mediated infiltration of Tregs (Huo et al., 2020; Wang et al., 2020)
	LMP1	Upregulates IL-10 (Yao et al., 1997; Ogino et al., 2007; Pai et al., 2007); Modulates PD-1/PD-L1 (Fang et al., 2014)
	LMP2A & 2B	Promote internalisation of INFRs (Shah et al., 2009)
	miR-BART6p	RIG-1 (Lu et al., 2017)
	miR-BART11, 17-3p	Promote *PD-L1* transcription (Wang et al., 2022)
	BZLF1/ Zta	Upregulates IL-10 (Lee et al., 2011)
Angiogenesis	EBERs	Upregulates *VCAM-1* (Cheng et al., 2019)
	EBNA1	Regulates AP-1 to upregulate IL-8, VEGF, HIF-1α (Oneil et al., 2008)
	miR-BART1-5p	Regulates the AMPK/mTOR/HIF-1 pathway (Wang et al., 2018)
Metastasis and invasion	BRLF1	Secretes MMP9 (Lan et al., 2018)
	EBNA1	Regulates TGF-β1/ ZEB / miR-200 feedback loop (Li et al., 2014b)
	LMP1	Regulates Snail and Twist (Horikawa et al., 2007, 2011); Enhances calreticulin expression (Ye et al., 2020); Induces hypermethylation of CDH1 (Tsai et al., 2002, 2006); Enhances the expression and activity of MMP9 (Takeshita et al., 1999)
	LMP2A	Mediates MTA1 (Lin et al., 2014); Regulates the integrin β4 (ITGβ4) subunit (Zhou et al., 2014; Liang et al., 2017); Enhances the expression of MMP9 (Lan et al., 2012)
	miR-BART7-3p	Targets PTEN (Cai et al., 2015)
	miR-BART8-3p	Targets RNF38 (Lin et al., 2018a)
	miR-BART9	Targets E-cadherin (Hsu et al., 2014)
	miR-BART10-3p	Targets BTRC (Yan et al., 2015)
	miR-BART13-3p	Targets ABI2 (Huang et al., 2020)
	miR-BART22	Targets MOSPD2 (Zhang et al., 2022)
Metabolic reprogramming	LMP1	Enhances GLUT1 transcription (Zhang et al., 2017); Induces FGFR1 expression and activation (Lo et al., 2015); Modulates c-Myc and HK2 (Xiao et al., 2014); Represses HoxC8 (Jiang et al., 2015)
	LMP2A	Downregulates adipose triglyceride lipase (Zheng et al., 2020)
	miR-BART1-5p	Activates the AMPK/mTOR/HIF1 pathway (Wang et al., 2018)

Several candidate genes that predispose to NPC were reported, including genes involved in immune responses. The genetic polymorphisms of the human leukocyte antigen (HLA) complex located at the major histocompatibility complex (MHC) region on chromosome 6p21 might confer susceptibility towards NPC due to impaired EBV antigen presentation to cytotoxic cells, hence contributing to ethnicity predisposition of EBV-associated NPC (Wee et al., 2012). Furthermore, given the male predominance in NPC incidences, it is hypothesised that the involvement of an X-chromosome-linked polymorphism, which affects the innate immune response, could enhance the susceptibility towards EBV infection. A possible candidate is a gene encoding the Toll-like receptor 8 (TLR8) which displays specific polymorphisms in East Asians (Wee et al., 2012). A next-generation sequencing technology-guided single nucleotide polymorphism (SNP) analysis revealed an association between SNPs of IL-10 and SPLUNC1 (short-palate lung and nasal epithelial clone 1; also known as BPI fold containing family A member 1) with increased susceptibility to NPC (Wu et al., 2017). In addition, whole-exome sequencing on multiplex families reported gene variants potentially involved in EBV viral infection, for instance, gene encoding integrin αvβ6 ITGB6 that facilitates EBV fusion to epithelial cells. BCL2L12 (B-cell lymphoma 2-like 12) and NEDD4L (Neural precursor cell expressed developmentally downregulated gene 4-like), which encode for LMP-interacting proteins, are also associated with familial NPC (Yu et al., 2019).

Exposure to carcinogens such as acetaldehyde from alcohol and polycyclic aromatic hydrocarbons from tobacco smoking induces the formation of DNA adducts, causing impairment of DNA repair and generation of reactive oxygen species that subsequently induce genetic alterations in the nasopharyngeal epithelium and results in the formation of low-grade dysplastic lesions that favours EBV infection and persistence (Basria et al., 2019). Particularly, reports on frequent loss of heterozygosity on chromosomes 3p and 9p in the normal nasopharyngeal epithelium of individuals with a high risk of developing NPC and in low-grade dysplastic lesions indicate that the genetic events take place during early pathogenesis of NPC and may precede EBV infection (Chan et al., 2000, 2002). The deletion of cyclin-dependent kinase inhibitor 2A (CDKN2A) on chromosome 9p leads to augmented expression of cyclin D1 (*CCND1*) and contributes to persistent latent EBV infection through suppressing EBV-induced growth arrest and senescence (Tsang et al., 2012). On the other hand, environmental factors, particularly cigarette smoking, are linked to EBV reactivation, which generates infectious products and modulates signalling cascades involved in carcinogenesis, contributing to NPC. Cigarette smoke extract promotes EBV replication and induces the expression of lytic genes *in vitro*. Epidemiological studies revealed the association of smoking with persistent reactivation of EBV, as reflected by elevated plasma levels of its antibodies, which increase the risk of carcinogenesis (Xu et al., 2012; Chen et al., 2022).

The clonal origin of EBV infection in NPC implies its involvement in the early initiation of NPC (Ajadurai et al., 1995; Lo et al., 2012). It is postulated that viral-induced epithelial changes occur at the transformational zone present in the fossa of Rosenmüller during early life when regulation of the host immune response relies on TLR8. Thus, EBV infection during early childhood potentially predisposes an individual to EBV-associated NPC (Poh et al., 2016). The exact mechanism for the entry of EBV into nasopharyngeal epithelial cells remains vague. Early studies suggested that EBV-specific polymeric IgA may induce a shift of EBV tissue tropism, facilitating its infection in epithelial cells. EBV bound to IgA may enter epithelial cells *via* endocytosis, a process mediated by the secretory component, a transmembrane protein expressed on the basolateral surface of epithelial cells (Sixbey and Yao, 1992; Lin et al., 1997). On the other hand, as demonstrated in both *in vitro* and *in vivo* models, it is surmised that EBV infection is established in nasopharyngeal epithelial cells primarily through cell-mediated infection (Yua et al., 2018). Studies also reported the involvement of integrins αvβ5, αvβ6 and αvβ8 in triggering epithelial cell fusion mediated by EBV glycoprotein gHgL (Chesnokova et al., 2009; Chesnokova and Hutt-Fletcher, 2011). Moreover, recent studies revealed two receptors that promote EBV internalisation and fusion with nasopharyngeal epithelial cells, including Neuropilin 1 (NRP1) and ephrin receptor tyrosine kinase A2 (EphA2; Wang et al., 2015; Zhang et al., 2018). The EBV infection in NPC is typically represented by a type II latency pattern, in which Epstein–Barr nuclear antigen 1 (EBNA1), latent membrane protein (LMP)1 and 2, and non-coding RNAs are expressed and induce further genetic and epigenetic changes, contributing to the acquisition of several cancer hallmarks. These hallmark capabilities, as enumerated by Hanahan and Weinberg, include [1] infinite proliferative potential; [2] evasion of growth suppressors; [3] resistance towards apoptosis; [4] immortal replication; [5] angiogenesis; [6] invasion and metastasis; [7] metabolic reprogramming and [8] elusion from immune destruction (Hanahan and Weinberg, 2011). The dysregulation of multiple signalling pathways induced by somatic mutations or EBV latent gene expression results in the modulation of the tumour microenvironment and immune response. The latent proteins further promote tumour progression by driving a rapid clonal expansion of EBV-infected cells, leading to the accumulation of genetic and epigenetic events, resulting in NPC progression (Lo et al., 2012; Tsang et al., 2012; Wong et al., 2021).

Several studies unveiled pieces of evidence of the involvement of the EBV lytic phase, characterised by the expression of EBV lytic genes in three stages, immediate early (IE), early (E), and late (L). in oncogenesis. The IE genes, *BZLF1* and *BRLF1*, are vital initiators of the lytic cascade that activate the E lytic genes that encode enzymes required for viral replication such as *BALF3*. Subsequently, L lytic genes are expressed and cell lysis takes place to release viral particles (Liang et al., 2022; Yap et al., 2022). Serological studies revealed the association between IE and E lytic genes with tumour progression and immune cell infiltration, suggesting the presence of intermittent abortive reactivation of EBV in NPC patients, the frequency whereof increases following disease progression (Guo et al., 2019). The lytic phase contributes to tumorigenesis by producing infectious virions that spread EBV infection to other cells and regulating cellular oncogenic pathways, including immune response, angiogenesis, invasion, apoptosis, and others (Rosemarie and Sugden, 2020).

### 2.1. EBV induces genetic instability in NPC

EBV induces genetic instability in infected premalignant cells through dysregulation of genes involved in [1] DNA repair; [2] cell cycle checkpoint; [3] anti-oncogenic activities. Through the PI3K/Akt/FOXO3a pathway, EBV-encoded LMP1 represses DNA damage-binding protein 1 (DDB1)-mediated nucleotide excision repair (Chen et al., 2008). Moreover, LMP1 induces G2 checkpoint defect by impairing Chk1 activation, allowing unrepaired chromatid breaks to undergo mitosis, which further propagates and accumulates, leading to chromosomal instability (Deng et al., 2012). On the other hand, EBV-encoded miRNAs, including *miR-BART5-5p*, *miR-BART7-3p*, *miR-BART9-3p*, *miR-BART14-3p*, modulate the activity of *ataxia telangiectasia mutated* (*ATM*) gene, which is involved in the repair of DNA double-strand break (Lung et al., 2018). EBNA1 is shown to promote the survival of cells harbouring DNA damage, contributing to NPC development. EBNA1 disrupts the promyelocytic leukaemia nuclear bodies (PML NBs) through interaction with cellular ubiquitin-specific protease (USP)7 or HAUSp, interfering with p53 acetylation, DNA repair and apoptosis (Sivachandran et al., 2008). EBV-encoded lytic gene product BALF3 could induce chromosome aberrations and DNA strand breaks in the host cells (Chiu et al., 2014).

EBV contributed to non-mutational genetic instability through the regulation of gene expression by inducing aberrant epigenetic alterations including DNA methylation and histone modification in NPC. A high frequency of hypermethylation was detected within or near the CpG islands (CGI) of malignant tissue, with significant enrichment of histone-bivalent marks at the methylated regions (Dai et al., 2015). EBV latent proteins, including EBNA1 and LMP1, could interact with DNA methyltransferases (DNMTs) or demethylases to modulate epigenetic modifications (Tsai et al., 2006; Shi et al., 2019). *Via* NF-κB signalling, LMP1 induces the hypermethylation and inactivation of the tumour suppressor phosphatase and tensin homologue (PTEN) by activating DNMT3b (Peng et al., 2016). DNA methylation and histone modification liaise to induce epigenetic deregulation in NPC. EBV infection dysregulates *MLH1*, a DNA repair pathway gene, *via* aberrant histone bivalent switches. The *MLH1* downregulation is associated with the reduced trimethylation of histone H3 lysine 4 (H3K4me3) and enhanced H3 lysine 27 trimethylation (H3K27me3; Leong et al., 2019).

### 2.2. EBV contributes to the construction of tumour microenvironment and immune evasion

The tumour microenvironment of NPC is featured by its abundance of immune cells, albeit with limited antitumour activities. EBV contributes to the accumulation of immune cells in the NPC microenvironment and immunosuppression in NPC, thus promoting tumour initiation, growth and development. Through CTAR1 and 2, LMP1 induces the upregulation of chemokines, including T-lymphocytes recruiting macrophage inflammatory protein (MIP)-1α and -1β, *via* NF-κB and JNK signalling cascades (Lai et al., 2010). EBV-encoded RNAs (EBERs) trigger inflammatory responses characterised by a rise in TNF-α levels in NPC *via* the TLR3 pathway. EBERs also collaborate with LMP1 through an NF-κB-mediated positive feedback loop to amplify the inflammatory response in NPC (Li et al., 2015).

EBV-encoded latent proteins and miRNAs contribute to viral persistent infection and tumorigenesis by compromising the host immune responses through the interference of cytokine signalling networks and antigen presentation, promoting the infiltration of immune-regulating cells in the NPC immune microenvironment, and inducing immune cells anergy. EBV modulates the innate immune response by the disruption of chemokine and cytokine signalling pathways. LMP2A and 2B modulate interferon (IFN) activity by accelerating the internalisation of IFN receptors (IFNRs; Shah et al., 2009). LMP1 and EBV lytic transactivator BZLF1/Zta induce the upregulation of immunosuppressive cytokines such as interleukin (IL)-10 that potentiates regulatory function in CD4^+^ T helper cells and reduces cytotoxic T-cell infiltration. IL-10 is positively associated with the expression of Fas ligand (FasL) expression, which might trigger Fas-mediated apoptosis in antitumour immune cells (Yao et al., 1997; Ogino et al., 2007; Pai et al., 2007; Lee et al., 2011). EBV-miR-BART6p targets the pattern recognition receptor retinoic acid-inducible gene (RIG)-I mRNA to inhibit EBV-triggered IFN-β response (Lu et al., 2017). EBV recruits regulatory immune cells into the NPC immune microenvironment, promoting immune evasion. EBNA1 promotes the accumulation of immunosuppressive Foxp3^+^ regulatory T-lymphocytes (Tregs) by driving C-C motif ligand (CCL)20- and transforming growth factor (TGF)-β-mediated infiltration of Tregs into the tumour site and stimulates the conversion of naïve T-lymphocytes into Tregs *via* upregulation of TGF-β and polarisation of M2 macrophages (Huo et al., 2020; Wang et al., 2020). Alteration of the metabolic pathway leads to the expansion of myeloid-derived suppressor cells (MDSCs) in the NPC microenvironment (Cai et al., 2017). EBV induces exhaustion in immune cells by upregulating co-stimulatory molecules. Through STAT3, AP-1, and NF-κB pathways, LMP1 modulates the immune checkpoint programmed cell death protein 1 (PD-1)/ programmed death ligand 1 (PD-L1), facilitating immune evasion (Fang et al., 2014). EBV-miR-BART11 and EBV-miR-BART17-3p enhance *PD-L1* transcription by inhibiting FOXP1 and PBRM1, respectively (Wang et al., 2022).

EBV drives angiogenesis in NPC, which is necessary for the transport of oxygen and nutrients to the malignant tissue. EBV infection induces store-operated Ca^2+^ entry (SOCE)-mediated angiogenesis through upregulation of its regulator, stromal interaction molecule 1 (STIM1; Ye et al., 2021). EBV-infected cells induce chemokine CCL5-mediated tumour angiogenesis *via* PI3K/AKT and HIF-1α pathways (Ma et al., 2018). Enhanced expression of transcription factor activator protein (AP)-1 by EBNA1 elevates the expression of angiogenic cytokine IL-8 and vascular endothelial growth factor (VEGF), leading to angiogenesis in NPC (Oneil et al., 2008). Exosome-packaged EBERs upregulate the expression of vascular cell adhesion molecule 1 (*VCAM-1*) expression to promote angiogenesis, the process involving TLR3 and RIG-I (Cheng et al., 2019). EBV-miR-BART1-5p induces angiogenesis by regulating the AMPK/mTOR/HIF-1 pathway (Wang et al., 2018). Besides classic angiogenesis, EBV induces vasculogenic mimicry, a process whereby malignant cells form vessel-like structures independent of endothelial cells. Activation of the PI3K/AKT/mTOR/HIF-1α signalling cascades by LMP2A promotes vasculogenic mimicry in NPC (Xiang et al., 2018). On the other hand, LMP1 induces the formation of vasculogenic mimicry by VEGF/VEGFR1 signalling (Xu et al., 2018).

### 2.3. EBV promotes metastasis and invasion

Metastasis is a complex process involving a series of events, including [1] invasion of tumour cells into adjacent tissues, [2] intravasation into the circulatory system, [3] survival in the peripheral blood circulation, [4] extravasation into distant tissues, and [5] colonisation (Lambert et al., 2017). Epithelial cells undergo epithelial-mesenchymal transition (EMT) to enhance their motility and invasiveness, the outcome marked by a decrease in epithelial markers such as E-cadherin and an increase in mesenchymal markers including vimentin, and morphological change into spindle-like shape. Studies revealed the prominence of cells with spindle-like morphology and expression of molecular markers associated with cancer stem cells and EMT at the invasive front of NPC tumours, these characteristics highly correlated with EBER and LMP1 (Luo and Yao, 2013). LMP2A was also reported to be associated with side population stem-like cancer cells, but not morphologic change (Kong et al., 2010). LMP1 promotes EMT through the regulation of transcription factors, including Snail and Twist (Horikawa et al., 2007, 2011). LMP1 increases the expression of calreticulin, promoting EMT *via* the TGF-β/Smad-3/NRP1 axis (Ye et al., 2020). Through the mTOR pathway, LMP2A induces the overexpression of metastatic tumour antigen 1 (MTA1) to promote EMT in NPC (Lin et al., 2014). By mediating the TGF-β1/ zinc finger E-box binding homeobox (ZEB)/ miR-200 feedback loop, EBNA1 induces EMT in NPC (Li et al., 2014b). EBV-miR-*BART7-3p*, *BART8-3p*, *BART10-3p*, *BART13-3p*, and *BART22* induce the EMT process by targeting *PTEN*, *RNF38*, *BTRC*, *ABI2* and *MOSPD2* genes, respectively, in NPC cells (Cai et al., 2015; Yan et al., 2015; Lin et al., 2018a; Huang et al., 2020; Zhang et al., 2022).

EBV targets cell adhesion molecules including cadherin and integrin to induce a mesenchymal-like phenotype of the host malignant cells. LMP1 downregulates the expression of E-cadherin by inducing hypermethylation of its gene promoter CDH1 (Tsai et al., 2002, 2006). EBV-encoded miRNA-BART9 elicits inhibitory action on E-cadherin, promoting the invasiveness property of NPC cells (Hsu et al., 2014). LMP2A promotes the motility of NPC cells by regulating the localization of the integrin β4 (ITGβ4) subunit at the cellular protrusions. LMP2A competitively bind to the spleen tyrosine kinase (Syk), which is responsible for the internalisation of the ITGβ4 subunit and inhibition of cell migration. Moreover, LMP2A accelerates the cleavage of ITGβ4 through the EGFR/ Ca^2+^/ calpain/ ITGβ4 pathway (Zhou et al., 2014; Liang et al., 2017).

Studies unveiled other metastatic mechanisms utilised by EBV, including the expression of matrix metalloproteinases (MMPs), which degrade the basement membrane and allow the invasion of NPC cells. LMP1 interacts with the tumour necrosis factor receptor family-associated factors (TRAFs) through CTAR-1 and CTAR-2, contributing to the activation of the MMP-9 promoter. It further induces the MMP-9 enzymatic activity through the activation of the NF-κB and AP-1 transcription factors (Takeshita et al., 1999). LMP2A triggers the expression of MMP9 through the ERK1/2-Fra-1 axis through its PY motifs (Lan et al., 2012). BRLF1/Rta promotes invasiveness in NPC through paracrine secretion of MMP-9 (Lan et al., 2018). Other than that, LMP1 upregulates neurotrophic tyrosine kinase receptor type 2 (NTRK2 or TrkB) expression to enhance anoikis resistance, which is necessary for survival after detachment from the tumour, of NPC cells through NF-κB signalling (Li et al., 2020).

### 2.4. EBV promotes metabolic reprogramming In NPC

The Warburg effect, or aerobic glycolysis, is commonly observed in malignant cells. EBV modulates the metabolic switch in NPC cells through the alteration of mitochondrial function, contributing to elevated glycolysis, enhanced proliferation, and apoptotic resistance (Xiao et al., 2014; Sung et al., 2017). LMP1 downregulates PTEN/AKT signalling through by upregulating the expression and activity of DNMT1 to activate the glycolytic flux. Furthermore, LMP1 promotes the mitochondrial translocation of DNMT1, thus inducing the hypermethylation of the mitochondrial DNA D-loop region and downregulation of oxidative phosphorylation (OXPHOS) complexes causing metabolic reprogramming in NPC (Luo et al., 2018). EBV is involved in the regulation of glucose metabolism through multiple signalling pathways. Through AKT/ERK/IKK signalling, LMP1 induces mTORC1 activation, which subsequently induces NF-κB signalling and enhances the transcription of glucose transporter (GLUT)1, thereby increasing glucose uptake in NPC cells (Zhang et al., 2017). Furthermore, LMP1 induces the expression of fibroblast growth factor receptor (FGFR)1 and activates its signalling, thus facilitating aerobic glycolysis through the phosphorylation of glycolytic pathway-associated proteins such as lactate dehydrogenase (Lo et al., 2015). BART1-5p activates the AMPK/mTOR/HIF1 pathway, facilitating the conversion to aerobic glycolysis (Wang et al., 2018). EBV modulates multiple transcription factors such as HIF-1α, c-Myc, and HoxC8 to alter glucose metabolism in NPC. LMP1 extends the half-life of c-Myc, a master regulator of energy metabolism, by attenuating the PI3K/Akt-GSK3β-FBW7 signalling axis, which mediates the ubiquitination-dependent degradation of c-Myc. C-Myc subsequently participates in LMP1-mediated upregulation of hexokinase (HK)2, a key player in glycolysis (Xiao et al., 2014). Through stalling RNA polymerase II, LMP1 represses Homeobox (Hox) genes, including a glycolytic regulator HoxC8 (Jiang et al., 2015). The shift in glucose metabolism facilitates tumorigenesis through its contribution to EBV-mediated immortalisation of infected cells, sustained EBV latency, apoptotic resistance and modulation of host immune response (Yang et al., 2022).

Besides glycolytic flux, metabolic shifts in lipids and amino acids were reported in NPC. LMP2A downregulates adipose triglyceride lipase, inhibiting lipid catabolism. This results in the accumulation of lipid droplets in NPC cells, thereby enhancing their migratory capabilities (Zheng et al., 2020). Plasma lipidomic revealed the association of plasma lipid profile with the titre of EBV antibodies reflecting the EBV lytic-latent cycle and with disease progression. Therefore, it is suggested that EBV potentially affect the prognosis of NPC by regulating lipid metabolism (Huang et al., 2022). Furthermore, NPC cells expressing LMP1 display enhanced consumption of glutamine (Lo et al., 2015).

The unique geographical and ethnic predominance of NPC highlights the importance of interplay among EBV, genetic susceptibility and environmental factors in the initiation and development of NPC. The advancement in multi ‘omic’ technologies has shed light on the understanding of the complex interactions in NPC carcinogenesis. The chemical carcinogen-induced genetic aberrations and inherited genetic susceptibility potentially enhanced individual predisposition towards EBV infection, while facilitating the establishment of latency and contributing to malignant alterations in host cells. Further elucidation of the precise pathogenic role of EBV in NPC would assist in the discovery of useful biomarkers and therapeutic targets. The development of more efficient *in vitro* systems for the investigation of the EBV life cycle in epithelial cells would allow more comprehensive studies of viral-host interaction in the oncogenesis of NPC. Future studies are required to define the biological role of the identified genes underlying genetic susceptibility towards NPC and their interactions with viral factors. The increase in sample size is suggested to obtain results with improved reliability and reproducibility, thus allowing the stratification of individual risk towards NPC and the development of cost-effective NPC screening strategies in high-risk populations. Furthermore, as environmental factors are associated with the development of NPC, the establishment of lifestyle modification programmes or guidelines targeting high-risk communities is recommended to minimise their risk of developing NPC. The strong association between EBV and NPC urges the development of prophylactic vaccines against EBV and validation of their long-term efficacy in preventing NPC, particularly in endemic regions.

## 3. EBV subtypes

Despite the widespread EBV infection which affects 90% of the global adult population, EBV-associated NPC displayed a skewed geographical distribution, suggesting the role of EBV sequence variation in NPC pathogenesis. This spurred studies into the geographical and ethnic correlation of various EBV subtypes and their role in the development of specific EBV-associated neoplasms. The major variation of the alleles at the EBNA2 and 3 s gene sequences clustered EBV into type 1 and type 2 strains. Type 1 EBV, including B95.8, Akata, Mutu, C666-1, M81, GD1, and GD2, displayed global prevalence and is commonly associated with NPC. On the other hand, type 2 EBV such as AG876, Jijoye, and Wewak is prevalent in Papua New Guinea, Alaska, and sub-Saharan Africa (Palser et al., 2015; Weiss et al., 2018).

EBV viral genomic analysis is imperative for the study of the association between viral sequence variation and EBV-associated diseases. Various EBV strains were isolated from NPC and fully sequenced, including two strains isolated from Cantonese NPC patients, Guangdong (GD)-1 (Zeng et al., 2005) and -2 (Liu et al., 2011), strains isolated from Hong Kong patients with NPC, HKNPC1-9 (Kwok et al., 2012, 2014) and M81 (Tsai et al., 2013), Yu103 (Yu et al., 2021a) from a Singaporean NPC patient, and an NPC cell line-derived C666-1 strain (Tso et al., 2013; Table 2). The EBV isolates exhibit distinct phenotypic properties, for instance, the M81 strain displays enhanced tropism towards epithelial cells and reduced tropism towards B-lymphocytes, which may be due to the abundance of the glycoprotein gp110 (Tsai et al., 2013). On the other hand, Yu103 simultaneously infects and transforms both B-lymphocytes and epithelial cells, thus possessing the capacity to drive both haematological and epithelial malignancies (Yu et al., 2021a).

**Table 2:** EBV strains isolated from NPC.

EBV genomes	Sources	Geographical region	GenBank accession number	References
GD1	NPC patient saliva	Guangdong	AY961628	Zeng et al. (2005)
GD2	NPC tumour biopsy	Guangdong	HQ020558	Liu et al. (2011)
HKNPC1	NPC tumour biopsy	Hong Kong	JQ009376	Kwok et al. (2012)
HKNPC2-9	NPC tumour biopsy	Hong Kong	KF992564KF992571	Kwok et al. (2014)
M81	NPC-derived EBV-infected lymphoblastoid cell line	Hong Kong	KF373730	Tsai et al. (2013)
C666-1	NPC cell line	Hong Kong	KC617875	Tso et al. (2013)
Yu103	NPC tumour biopsy	Singapore		Yu et al. (2021a)

The latency-associated genes possess the highest frequency of SNP (Palser et al., 2015). Notably, the LMP1 gene sequence displays a high degree of polymorphism when compared with other EBV genes. These include a 30 base-pair (bp) deletion at the cytoplasmic carboxyl (C)-terminal tail, which is associated with a more aggressive phenotype of EBV-associated tumour. It is reported that a 30 bp deletion results in codon variation of the T-cell epitope, leading to potential escape from host cell immune recognition. Furthermore, a point mutation involving a single nucleotide substitution at position 169,425 results in the loss of the XhoI restriction site of the cytoplasmic amino (N)-terminal tail. These mutations are suggested to be associated with NPC susceptibility (Lin et al., 2003; Tang et al., 2008; da Costa et al., 2015; Banko et al., 2021). The LMP1 gene heterogeneity defines several variants, which are Alaskan, China 1, China 2, China 3, Mediterranean (Med)+, Med-, and North Carolina (NC; Edwards et al., 1999).

The polymorphisms at the codon 487 of EBNA-1 at the C-terminal give rise to 5 subtypes, including two prototypes P-Ala and P-Thr and three variants V-Val, V-Leu, and V-Pro. The V-Val subtype, which is commonly detected in NPC biopsies from Asian patients, might contribute to NPC tumorigenesis by enhancing the expression of viral or cellular genes (Zhang et al., 2004; Van Do et al., 2008; Mai et al., 2010; Lha et al., 2019). NPC-derived EBNA1 genes typically possess polymorphisms in their DNA-binding domain, leading to defective DNA replication and episome maintenance that potentially increases NPC risk due to unstable latent infection and aberrant expression of lytic cycle genes (Dheekollu et al., 2017).

Variations in the EBV lytic gene contribute to increased NPC risk. The *BALF2* H-H-H endemic strain carrying three non-synonymous single nucleotide variants (BALF2 162215A > C [V700L], 162,476 T > C [I613V], and 163364C > T [V317M]) in the EBV genome is associated with NPC in southern China. The SNPs 162,476_C and 163364_T confer an increase in risk exceeding 6-fold for NPC and cumulatively contribute to 83% of overall NPC risk in southern China (Xu et al., 2019; Miller et al., 2021; Kondo et al., 2022). Furthermore, the amino acid substitution in V1222I of BNRF1 is found to be associated with NPC risk in China. The variations in *BALF2* and *BNRF1* may result from viral interaction with the host immune system (Xue et al., 2021).

Recent studies identified several polymorphisms associated with EBV risk within the EBV genome. These include a single nucleotide polymorphism within the EBV-encoded *RPMS1* gene (G155391A) which was reported to be associated with a high risk of NPC (Feng et al., 2015). Further investigation of the *RPMS1* gene polymorphisms revealed four genotypes, RPMS1-A to D, whereby the variation is correlated with geographical region and type of malignancy (Wu et al., 2018). Other than that, a four-base deletion within the EBER locus was detected in approximately 97% of NPC cases (Fung Hui et al., 2018).

Viral genetic variation potentially elicits an influential effect on NPC carcinogenesis, though the relationship between EBV genetic variation and the risk of NPC is yet to be established. Furthermore, the majority of studies on EBV genotyping were limited to certain regions. Moreover, it was reported that EBV sequence variation is associated with host sequence variants, hence, joint analyses of host and viral genome variation would help decipher the pathogenesis of NPC and identify novel diagnostic and therapeutic targets (Rüeger et al., 2021). Studies recruiting larger samples involving individuals with different conditions from different ethnic groups and geographical regions are needed to determine the exact association of EBV strains with the geographical distribution and development of the disease. Further characterisation of mutations in the high-risk EBV variants would facilitate the delineation of molecular mechanisms that take place in the infected host cells. The implementation of new techniques, such as clustered regularly interspaced short palindromic repeats (CRISPR)/Cas9 system would allow rapid cloning and sequence determination of EBV variants (Yajima et al., 2018).

## 4. EBV-based biomarkers in NPC

The insignificant clinical manifestations of NPC, including otologic conditions, neck mass, nasal issues, and headache, and its anatomic isolation led to delayed diagnosis and treatment, whereby patients only start seeking medical attention 6 weeks after the onset of symptoms. A misdiagnosis rate of as high as 43.4% was reported in NPC patients (Wu et al., 2016; Wang et al., 2017). Based on the AJCC/IUCC’s 8^th^ edition TNM staging system for NPC, the five-year OS for NPC patients diagnosed at early stages (stages I and II) are more than 90%, whereas in patients diagnosed with advanced stages NPC, the five-year OS decline drastically, whereby a 5-year OS of 73.7% is predicted in patients with stage IVA NPC (Jen et al., 2020). Thus, early diagnosis of NPC is essential to improve the survival and prognosis of patients. Nasoendoscopy examination combined with pathologic analysis of biopsy remains the current gold standard for clinical NPC detection. The approach is however invasive and not suitable for asymptomatic individuals (Zhang et al., 2021b). This warrants the development of non-invasive tests for the diagnosis of NPC which offers high specificity, sensitivity, accuracy and reliability. Given the close association between EBV and NPC, EBV represents a promising target for the development of screening, diagnostic and prognostic biomarkers for NPC.

A two-stage screening model is used for population screening in endemic regions. The model consists of a primary screening method using serological or molecular markers, followed by secondary screening using imaging or endoscopy methods. Conventionally combined detection of VCA/ IgA and EA/IgA using immunoenzymatic assay was performed in endemic areas of NPC, allowing the early diagnosis of NPC with a rate ranging from 55 to 87% (Zeng et al., 1980, 1982; Ji et al., 2002; Lo et al., 2022). The increased titres of EBV-specific IgA and IgG antibodies in the serum of NPC patients, as first reported in the 1970s, provide a rationale for the intervention (Henle and Henle, 1976; Ho et al., 1976). Studies revealed the presence of a serologic window wherein serological EBV-VCA IgA antibody levels are elevated and sustained which precedes the clinical onset of NPC, suggesting EBV-VCA IgA as an early marker of NPC (Ji et al., 2007). The development of the ELISA technique for the detection of multiple EBV serological markers, including immunoglobulins to VCA, EA, EBNA1, Zta, and replication and transcription activator (Rta), aims to improve the accuracy, sensitivity and specificity of the screening assay. Plasma EBNA1-IgA demonstrates a remarkable distinguishing ability between early-stage NPC and normal subjects with specificity and sensitivity as high as 98.7 and 91.5%, respectively. Combined detection of VCA-IgA with EBNA1-IgA by ELISA enables the diagnosis of NPC patients with improved specificity and sensitivity (Fachiroh et al., 2006; Liu et al., 2012, 2020).

By using conventional PCR targeting EBNA, Mutirangura et al. detected the presence of EBV DNA, which might be originated from NPC tumour, in 31% of blood samples obtained from NPC patients and suggested its potential as a marker for the diagnosis of NPC (Mutirangura et al., 1998). Case–control studies employing the real-time PCR (qPCR) strategy to target the BamHI-W and EBNA1 regions of the viral genome and detected cell-free EBV DNA in over 95% of plasma from NPC patients (Lo et al., 1999; Lin et al., 2004). Large-scale screening of high-risk populations combining qPCR-based detection of EBV DNA, nasal endoscopic examination and magnetic resonance imaging (MRI) allowed the identification of early-stage NPC, with specificity and sensitivity of 98.6 and 97.1%, respectively, (Lam et al., 2017). The sensitivity of EBV DNA for the detection of early-stage NPC (90%) was limited when compared to advanced-stage NPC (98%), implying the need for parallel screening to enhance diagnostic sensitivity. For instance, the combination of EBV DNA and IgA-VCA as marker panels could enhance the diagnostic sensitivity to 99% (Leung et al., 2004). Moreover, high levels of EBV DNA load can be detected in the nasopharyngeal swab of NPC patients with a lower rate of false-positive and -negative when compared to plasma EBV DNA, suggesting nasopharyngeal EBV DNA as an alternative with less invasiveness and higher accuracy than plasma EBV DNA (Zheng et al., 2015a). Other than that, sequencing-based detection of plasma EBV DNA displayed superior positive predictive value over qPCR and was able to distinguish the molecular characteristic of circulating EBV DNA between NPC and non-NPC individuals, thus reducing false positives for the diagnosis of NPC (Lam et al., 2018).

The plasma EBV DNA concentration is correlated with the disease stage, clinical outcome and overall survival of NPC patients, indicating its potential as a prognostic indicator for staging and monitoring of therapeutic effect (Lo et al., 1999; Lin et al., 2004). Patients with undetectable post-treatment plasma EBV DNA had better survival, while the presence of EBV DNA in plasma following treatment predicts distant metastasis. It is suggested that the integration of EBV DNA with other clinicopathologic parameters before, during, and after treatment could facilitate outcome prediction, treatment strategy tailoring, response monitoring, and post-treatment surveillance. Prognostic nomograms incorporating EBV DNA load and the UICC/AJCC staging system were established for prognosis prediction and treatment stratification in patients with locally advanced NPC (Xu et al., 2017). A recent large-scale study established a recursive partitioning analysis model that classified elderly patients with NPC into risk groups based on EBV DNA load and T stage and the chemotherapy benefits in each group were assessed. In the poor prognosis group, defined by EBV DNA load of fewer than 4,000 copies/mL with stage T3–4 or EBV DNA of more than 4,000 copies/mL with any T stage, patients receiving chemoradiotherapy had significantly improved OS compared with patients receiving only radiotherapy (Wu et al., 2022). Pre- and post-treatment plasma EBV DNA titres were predictive of the risk of disease progression, metastasis, recurrence, and death, and thus are promising biomarkers for prognosis stratification and post-treatment tumour surveillance (Li et al., 2021). Nevertheless, the heterogeneity of the assay methodologies and lack of a standardised reference system leads to interlaboratory discrepancies. Thus, standardisation of methods and cut-off values for EBV DNA assays is required to ensure reproducibility and improve the efficacy of the marker (Lee et al., 2021).

Besides that, EBV-encoded miRNAs emerge as potential diagnostic and prognostic biomarkers of NPC. miRNA profiling using qPCR detected the elevation of a series of EBV-encoded BART miRNAs, including BART2-5p, BART6-3p, BART7-3p, BART7-5p, BART9-5p, BART11-3p, BART17-5p, and BART19-5p in the plasma samples of patients with primary NPC, wherein BART19-5p displayed the best performance for the detection of NPC in patients with undetectable sera EBV DNA (Gao et al., 2019). Sera miRNA BART2-5p allows the identification of preclinical NPC from high-risk populations with 90.9% sensitivity and 54.5% specificity (Jiang et al., 2018). Moreover, enhanced plasma levels of BART2-3p, BART2-5p, BART5-3p, BART5-5p, BART6-3p, BART8-3p, BART9-5p, BART17-5p, BART19-3p, and BART20-3p were associated with recurrent NPC, among which BART8-3p and BART10-3p demonstrated the highest ability to distinguish EBV DNA undetectable recurrent NPC (Gao et al., 2019). Abundant levels of miRNAs BART7 and BART13 are detected in the plasma samples from NPC patients, the levels increase with disease progression. miRNAs BART7 and BART13 might serve as prognostic biomarkers that provide useful clinical information on disease stage and treatment efficacy (Zhang et al., 2015; Lu et al., 2020). Large-scale studies should be conducted to further validate the efficacy of EBV-encoded miRNAs as biomarkers in NPC.

To facilitate the integration of these biomarkers into clinical use, the method and cut-off value should be standardised to produce accurate and reproducible results. Moreover, large-scale clinical studies with consideration of geographical, genetic, and environmental factors should be conducted. Research on big data analytics would shed light on the disease pattern and its associated factors. The integration of patient information from the aspect of genomics, proteomics, imaging, serological profile, demographics and others allows for the tailoring of a personalised treatment strategy for each individual. Artificial intelligence is developed to provide insights into intervention strategies for precision medicine. Researchers have developed machine learning and deep learning models for the prediction of survival, disease progression, and treatment response for NPC patients by integrating several clinical parameters, including images, demographic characteristics, plasma EBV-DNA, serum biochemical profile, treatment regimen and others (Jing et al., 2019; Zhong et al., 2021; Chen et al., 2021a; Zhang et al., 2021a; Ng et al., 2022).

## 5. Ongoing clinical and preclinical studies on therapeutic targeting EBV in NPC

Over the last few decades, advances in the management of NPC have brought about a significant improvement in the prognosis of patients. The current treatment strategies for NPC patients, as outlined in the National Cancer Comprehensive Network (NCCN) guideline (v 1.2018) and ESMO-EURACAN guideline, are mainly based on AJCC/IUCC staging. IMRT is the current benchmark for the management of early-stage NPC (stage I-II), with locoregional control exceeding 90%. Unfortunately, nearly 70% of patients are presented with locoregionally advanced disease upon diagnosis, leading to poor outcomes. The addition of concurrent cisplatin chemotherapy to radiation in patients with advanced disease (stage III-IVb) significantly improves the prognostic outcomes (Au et al., 2018; Colevas et al., 2018; Bossi et al., 2021; Yang et al., 2021). Despite remarkable improvement in the clinical benefit of NPC patients, 5 to 15% of patients receiving IMRT will develop locoregional failures, while 15 to 30% of patients will experience distant failure. Furthermore, patients also suffer from late toxicities, such as neurologic damage and hearing loss (Lee et al., 2015). The development of effective therapeutic strategies which address specific molecular targets of NPC would improve the efficacy of cancer therapy while reducing its toxicity and cost. The presence of EBV in undifferentiated NPC cases and premalignant lesions imply the significant role of EBV in the development of NPC. Given the oncogenic role of EBV in NPC, studies have exploited various approaches targeting EBV, including immunotherapy, lytic reactivation, latent proteins-targeting peptides, and genetic therapy (Table 3).

**Table 3:** Clinical studies on EBV-targeting therapeutics for NPC. R/M NPC: recurrent/ metastatic NPC.

Reference	Study	Conditions	Phase
**Immunotherapy**			
NCT02578641	Combined gemcitabine-carboplatin (GC) & adoptive T-cell therapy	Advanced NPC	III
NCT02287311	LMP, BARF1 & EBNA1 specific CTL	EBV-positive tumours	I
NCT03648697	EBV-TCR-T (YT-E001) cells	R/M NPC	II
NCT01094405 (Hui et al., 2013a)	MVA EBNA1/LMP2 vaccine	R/M NPC	II
NCT01800071 (Taylor et al., 2014)	MVA EBNA1/LMP2 vaccine	R/M NPC	Ib
**Genetic therapy**			
NCT01449942 (Liao et al., 2014)	LMP1-DNAzyme	NPC	I
**Cytolytic Virus Activation Therapy**			
(Stoker et al., 2015)	Gemcitabine, valproic acid & ganciclovir	R/M NPC	I/II

### 5.1. Immunotherapy

Cellular-based immunotherapy targeting EBV in NPC can be further classified into two approaches, passive immunotherapy or adoptive immunotherapy, and active immunotherapy or cancer vaccines. Adoptive immunotherapy involved the infusion of EBV-targeting cytotoxic T-lymphocytes (CTLs) into NPC patients. Autologous EBV-specific effector T-cells include polyclonal T-cell lines reactivated *ex vivo* using autologous EBV-transformed lymphoblastoid cell line (LCL) and T-cells selectively reactivated using recombinant viral vectors or peptides. Phase I/II clinical studies reported the safety and well-tolerance of immunotherapy involving the adoptive transfer of autologous EBV-CTLs generated *ex vivo* by stimulation with EBV-transformed LCLs, albeit with limited efficacy (Straathof et al., 2005; Louis et al., 2010; Chia et al., 2014). Following infusion, the patients displayed a reduction in EBV viral load and improved response to chemotherapy. Patients with advanced NPC administered with chemotherapeutic agents, gemcitabine and carboplatin, combined with adoptive transfer of EBV-CTL demonstrated improved clinical outcomes when compared with patients receiving only chemotherapy, with an improved median overall survival from 17.7 months to 29.9 months, and an increase in 2-years OS rates from 29.5 to 62.9% (Chia et al., 2014). A phase III clinical study (NCT02578641) is currently being conducted across Asia and the United States to further evaluate the safety and efficacy of combined EBV-CTL adoptive immunotherapy with gemcitabine-carboplatin chemotherapy. Although potent antitumour activity was observed in patients with locoregional disease, patients with metastatic disease displayed only limited antitumour activity (Louis et al., 2010). Furthermore, the approach preferentially expanded EBNA3-specific T-cells, which are not expressed in NPC (Straathof et al., 2005). The generation of LCL-expanded EBV-specific CTLs is time-consuming, taking about 8 to 22 weeks, and labour-intensive (Chia et al., 2014).

Alternatively, EBV-specific CTLs are generated by stimulation with adenovirus-based vector encoding truncated EBNA1 fused to CTL epitopes from LMP1 and 2 (AdE1-LMPpoly). The method is rapid and generates T-cells with improved functional and cytolytic potential compared to LCL-expanded EBV-specific CTLs. In both prophylactic and therapeutic settings, adoptive immunotherapy with AdE1-LMPpoly-expanded T cells was well-tolerated in NPC patients. Patients receiving CTLs have a prolonged median OS of 523 days when compared to patients who did not receive CTLs (220 days). The immune response was however transient, and only a minimal decline in EBV DNA load was observed (Smith et al., 2012, 2017). Another approach involves genetic modification of T-cells to recognise tumour-specific antigens through the expression of chimeric antigen receptors (CAR). LMP1-targeting CAR-T cells displayed LMP1-specific cytolytic action and significant growth inhibitory effect on tumour overexpressing LMP1 in *in vivo* xenograft model (Tang et al., 2014). On the other hand, T-cell receptor (TCR) gene transfer has been exploited as an alternative approach with improved efficiency, reliability and reduced time consumed to generate a large number of T-cells targeting EBV-encoded oncoproteins with high avidity. A significant reduction of tumour growth in mice was observed (Zheng et al., 2015b). An ongoing phase II clinical study (NCT03648697) in China is currently being conducted to evaluate the efficacy of EBV-TCR-T (YT-E001) cells in NPC patients.

Adoptive immunotherapy targeting EBV is a promising therapeutic strategy for NPC with minimal toxicities and long-term clinical benefits. Several strategies are proposed to improve the quality and efficacy of the therapeutic approach. This include the discovery of predictive biomarkers such as IP-10 and MIP-3a and prognostic biomarkers such as EBV-DNA would facilitate in identifying patients who can benefit from the therapeutic plan (Chia et al., 2014). The limited expansion of EBV-specific CTLs *in vivo* might restrict the antitumour activities. CD45 monoclonal antibody (mAb)-mediated lymphodepletion could stimulate the expansion of infused EBV-specific CTLs through elevating plasma levels of cytokines IL-7 and 15, depletion of regulatory T-cells and increasing antigenic stimulation (Louis et al., 2009). The expression of co-inhibitory receptors such as PD-1 and CTLA-4 on EBV-specific CTLs might be associated with their limited antitumour cytotoxicity, hence, immune checkpoint blockade might help potentiate the therapeutic efficacy (Smith et al., 2017). The lack of appropriate animal models of NPC for the evaluation of the therapeutic potential of EBV-targeted immunotherapy is a major hurdle for its preclinical validation, leading to disparity in preclinical and clinical results. This highlights the need for preclinical models which recapitulate the human immune system and tumour microenvironment.

Apart from that, tumour vaccines delivering EBV-specific antigens, including LMP1, LMP2, and EBNA1, through viral vectors or antigen-presenting cells are designed to enhance tumour-specific immunity. MVA-EL, a recombinant Modified Vaccinia Ankara expressing a chimeric antigen construct composed of HLA class I-restricted epitopes in LMP2 and HLA class II-restricted epitopes in EBNA1, efficiently elicits CD8^+^ and CD4^+^ T-cells responses *in vitro*. In phase I clinical trials involving NPC patients in remission or with persisting diseases in Hong Kong and the United Kingdom, the vaccine was reported to demonstrate satisfactory safety and tolerability, and immunogenicity across diverse EBV strains and HLA variations (Taylor et al., 2004, 2014; Hui et al., 2013a). *In vitro* study suggested that LMP2-specific CTLs induced by EBV-LMP2 recombinant adenovirus vaccine elicit cytotoxicity effect on tumour cells *via* the perforin/ granzyme and Fas/FasL pathways (Ge et al., 2020).

The other strategy involves autologous antigen-presenting cells expressing EBV-specific antigens generated *ex vivo*. A dendritic cell (DC) vaccine generated by transduction with an adenovirus encoding a truncated LMP1 and full-length LMP2 (Ad-ΔLMP1-LMP2) displayed only limited efficacy in phase II clinical trials on metastatic patients (Chia et al., 2012). Another LMP2-DC vaccine, generated by infecting autologous DCs with an adenovirus vector expressing LMP2 (rAd-LMP2), can elicit CTL responses specific to LMP2 that contribute to preventing the recurrence and metastasis, particularly in the early stage of NPC (Zeng et al., 2020).

Tumour vaccines targeting EBV-encoded products are well tolerated across diverse ethnicity and have minimal adverse events. Limited efficacy was however reported in patients with advanced NPC, which may be due to the prevalence of Tregs and the presence of co-inhibitory molecules in the tumour microenvironment (Chia et al., 2012; Hui et al., 2013a). Future multicentre clinical trials recruiting larger cohorts are required to further assess the efficacy of these vaccines for the treatment of NPC, identify groups that could benefit from the intervention, and evaluate the long-term therapeutic effects of EBV-targeting vaccines. Studies should also investigate the potential synergism between therapeutic vaccination and other modalities, such as chemotherapy and adoptive immunotherapy to improve their efficacy. Virotherapy using oncolytic viruses represents a novel form of cancer therapeutics that act as cancer vaccines which stimulate the host’s anti-tumour immunity besides directly inducing cancer cell death. These include herpes simplex virus G47Δ and Pteropine Orthoreovirus 7S which demonstrated antitumour activity against NPC *in vitro*. Oncolytic viruses have been genetically engineered to express cytokines, costimulatory molecules, immune checkpoint inhibitors, or bispecific T-cell engager (BiTE) molecules to boost anti-tumour immune response and suppress tumour tolerance. For instance, an oncolytic adenovirus MG1 encoding HPV oncogenes E6 and E7 demonstrated tumour-specific responses in preclinical studies and is currently undergoing a phase I clinical trial involving patients with HPV-associated malignancies (Wang et al., 2011; Loh et al., 2022; Tan et al., 2022). Thus, oncolytic viruses represent attractive targets for the development of therapeutic tools against EBV-associated NPC.

### 5.2. Cytolytic virus activation therapy

Cytolytic virus activation therapy aims to reactivate the lytic cycle in latent EBV, thus inducing the expression of immunogenic proteins and viral kinases that sensitises cells to antiviral immune responses or therapies. The chemical lytic inducers involved include conventional chemotherapeutic agents, histone deacetylase inhibitors, proteasome inhibitors, protein kinase C (PKC) modulators and others (Wildeman et al., 2012). Chemotherapeutic drugs, including cis-platinum and 5-fluorouracil, induce a latent-lytic switch in EBV infecting malignant cells through p38 stress MAPK, P13 kinase, and PKC-δ pathways, thereby triggering *de novo* expression of viral kinases such as protein kinase (PK) that converts analogue anti-viral prodrug ganciclovir into active cytotoxic form to kill EBV-positive NPC cells (Feng et al., 2002; Meng et al., 2010). Inhibitors of histone deacetylase such as suberoylanilide hydroxamic acid (SAHA), or Vorinostat, and romidepsin, induce EBV lytic cycle with the expression of lytic proteins, leading to apoptosis and suppression of tumour growth in NPC (Hui et al., 2012, 2016). Sodium phenylbutyrate (NaPB), a PKC modulator, can upregulate EBV thymidine kinase (TK) activity in EBV-positive tumour cells, sensitising them to the antiviral drug ganciclovir (Chung et al., 2000). Iron chelators, including C7, and di-2-pyridyl ketone 4,4-dimethyl-3-thiosemicarbazone (Dp44mT) reactivate EBV lytic cycle through the extracellular signal-regulated kinase (ERK)-mediated autophagy and hypoxic signalling through HIF-1α induction (Choi et al., 2015; Yiu et al., 2018). Studies reported synergistic effects in the combination of chemical lytic inducers. For instance, the chemotherapeutic agent gemcitabine with histone deacetylase inhibitor valproic acid leads to stronger induction of the lytic cycle of EBV (Novalić et al., 2017). The combination of proteasome inhibitor bortezomib and SAHA synergistically activates EBV lytic cycle and induces ROS-driven caspase-dependent apoptosis, thus suppressing tumour growth *in vivo* (Hui et al., 2013b).

Pilot and phase I-II clinical studies reported that cytolytic virus activation therapy combining gemcitabine, valproic acid, and ganciclovir was well tolerated and contributed to disease stabilisation in patients with advanced NPC. Immune responses were restricted, probably due to the immunomodulatory effect of the tumour microenvironment (Wildeman et al., 2012). Future studies should look into the exact molecular mechanism underlying tumour and immune responses following lytic induction to enhance the efficacy of the therapeutic strategy. The effect of intracellular iron chelation suggested the incorporation of clinically available iron chelators for NPC patients. Further clinical studies would aid in the evaluation of the optimised dose and drug combination regimen in NPC patients.

### 5.3. Others

Peptides targeting EBV-encoded latent proteins were designed for cancer monitoring and precision cancer therapy. A probe combining the fluorophore L2 and EBNA1-specific binding peptide P4 was synthesised as a theragnostic agent that permits the visualisation of tumours when localised at the nucleus of EBV-positive NPC cells and inhibition of tumour growth by interfering with the homodimerization of EBNA1 (Jiang et al., 2017). The incorporation of a zinc chelator (ZRL5) into P4 could interrupt the oligomerization of EBNA1 more effectively to inhibit growth and reactivate EBV lytic cycle, leading to shrinkage of EBV-positive NPC tumours (Jiang et al., 2019). In another study, the conjugation of P4 with the lanthanide upconversion nanoparticles to form UCNP-P4 further enhances its stability and biocompatibility and provides stronger signalling for imaging and more specific targeting of tumours (Zha et al., 2018). The construction of a dual-EBV-oncoproteins-targeting pH-responsive peptide, UCNP-P5 further enhanced the specificity and efficacy of the drug while reducing the undesired side effects in preclinical *in vivo* and *in vitro* models (Zha et al., 2021).

Genetic manipulation of LMP1 expression *via* RNA-cleaving deoxyribozyme (DNAzyme) has been actively investigated as a potential treatment approach for NPC. DNAzymes showed an inhibitory effect on the expression of LMP1 and its downstream pathways, including the AP1, NF-κB and JAK/STAT pathways, which results in DNA damage and cell cycle arrest, promotion of apoptosis, and enhancement of radiosensitivity in both *in vitro* and *in vivo* NPC models. LMP1-targeted DNAzymes contribute to the inhibition of LMP1-induced radio-resistance in NPC by repressing the LMP1/JNKs/HIF-1/VEGF-mediated angiogenesis and inhibiting the LMP1/Akt-induced telomerase activity (Ma et al., 2013; Yang et al., 2014, 2015). Early phase clinical trial of intratumoral administration of EBV-LMP1 targeted DNAzyme in conjunction with radiotherapy reported a significant reduction in short-term tumour regression with minimal adverse events associated with the combined treatment (Cao et al., 2014; Liao et al., 2014).

Several strategies targeting EBV have been proposed and experimented with over the last few decades, including adoptive immunotherapy and cancer vaccines, cytolytic virus activation therapy, EBV-encoded oncoproteins-targeting peptides, and genetic therapy. Besides the widely explored EBNA1 and LMPs, the inevitable oncogenic roles of EBV-derived RNAs, including EBERs, miRNAs urged further investigation as promising therapeutic targets. NPC patients respond to EBV-targeting treatment variably, thus, the identification of predictive molecular markers is vital for the stratification of personalised treatment strategies for individual patients. This is however hindered by the limited availability of reliable NPC models which possess stable genetic aberrations. Therefore, patient-derived NPC models which are more genomically characterised are demanded to unravel the specific molecular players that determine patients’ responses to EBV-targeting therapy (Lee et al., 2015). Besides that, EBV is also an attractive target for tumour-specific drug delivery, which could enhance the potency of drugs while minimising undesired adverse effects in patients. Besides the aforementioned probed nanoparticles, exosomes are also promising vehicles for tumour-specific drug delivery (Wan et al., 2020). Genomic editing tools, such as CRISPR-Cas potentially mediate precise targeting of the viral genome for antitumour therapy (Huo and Hu, 2019). Lastly, studies combining different therapeutic strategies are also recommended to study their synergistic effect and design an optimal therapeutic regimen for NPC patients.

## 6. Concluding remarks and future opportunities

EBV is an oncogenic virus closely associated with NPC. The role of EBV infection in the initiation and development of NPC may be due to the aberrant induction of latency in premalignant epithelial cells, the exact molecular mechanisms remained unexplored. The preclinical research on the EBV association with NPC is impeded by the limited availability of representative models. Rapid loss of EBV episomes following cell propagation and genetic contamination was reported in cell cultures of NPC (Dittmer et al., 2008). The addition of ROCK inhibitor Y-27632 to epithelial cells suppressed their differentiation and promotes the establishment of EBV-positive cell line NPC43 (Lin et al., 2018b). The establishment of more efficient *in vitro* models for the investigation of EBV infection and cell transformation would offer insight into the interaction between host cells, viruses and the tumour microenvironment. Furthermore, patient-derived xenografts could recapitulate the mutational profile of NPC patients and facilitate the translational research and development of precision medicine.

NPC displayed skewed geographical and ethnic distribution despite the widespread EBV infection, suggesting the role of EBV gene heterogeneity. The implementation of more sensitive and effective technology for viral genome sequencing and cloning of EBV strains, together with phenotypic investigation of EBV sequence heterogeneity would shed light on identifying the high-risk EBV variant associated with NPC and the role of sequence variation in tumorigenesis, cancer progression, survival outcome, and therapeutic response. This knowledge would also facilitate the identification of high-risk groups with a predisposition to NPC and the development of prevention strategies or EBV-based therapeutics for NPC.

EBV serology detecting levels of EBV DNA and antibodies towards EBV oncoproteins aids in the detection of early-stage NPC, prediction of prognosis, and monitoring of patient’s response. The development of diagnostic and predictive biomarkers with high sensitivity and specificity is essential to detect NPC at an early stage and provide prognostic information with higher accuracy and precision. Large-scale, international studies with standardised methods and cut-off values are required to improve the efficacy of the markers. Studies integrating EBV-based signature with other NPC biomarkers, for instance, NPC-associated genomic polymorphisms, are required to further enhance their efficiency in early diagnosis and predict the treatment response and recurrence risk. The use of artificial intelligence integrating the genotypic and phenotypic profiles of patients could facilitate risk stratification and decision-making in a cost- and time-effective manner.

## Author contributions

ZS, PS and S-CC: conceptualization. ZS: writing—original draft preparation. PS, C-OL and S-CC: writing—review and editing. S-CC: funding acquisition and supervision. All authors contributed to the article and approved the submitted version.

## Funding

This study is supported by the Ministry of Higher Education Malaysia (MOHE) through Fundamental Research Grant Scheme FRGS (FGRS/1/2020/SKK0/UCSI/02/2).

## Conflict of interest

C-OL was employed by AGTC Genomics Sdn Bhd.

The remaining authors declare that the research was conducted in the absence of any commercial or financial relationships that could be construed as a potential conflict of interest.

## Publisher’s note

All claims expressed in this article are solely those of the authors and do not necessarily represent those of their affiliated organizations, or those of the publisher, the editors and the reviewers. Any product that may be evaluated in this article, or claim that may be made by its manufacturer, is not guaranteed or endorsed by the publisher.
